# Real-Time Breath Diagnostics: Linking Molecular Pathways, Measurement Technologies, and Clinical Translation

**DOI:** 10.3390/ijms27104276

**Published:** 2026-05-11

**Authors:** Velmurugan Thavasi, Nirmal Choradia, Naoko Takebe, Neal Naito, Susan Yeyeodu, Peter William Sadler, Dean Hougen, Sanchith Velmurugan, Jordan P. Metcalf, Donna L. Tyungu, Thirumalai Venkatesan

**Affiliations:** 1 Department of Physics & Astronomy, University of Oklahoma, Norman, OK 73019, USA; 2 OU Health Stephenson Cancer Center, 800 NE 10th St., Oklahoma City, OK 73104, USA; 3 MVK Pharmaceutical LLC, 31800 N Capitol Ave, Indianapolis, IN 46202, USA; 4 School of Biological Sciences, University of Oklahoma, Norman, OK 73019, USA; 5 School of Computer Science, University of Oklahoma, Norman, OK 73019, USA; 6 School of Electrical and Computer Engineering, University of Oklahoma, Norman, OK 73019, USA; 7 Pulmonary, Critical Care & Sleep Medicine Division, Department of Medicine, University of Oklahoma Health Campus, 800 N. Research Parkway, Oklahoma City, OK 73104, USA; 8 Pediatric Infectious Diseases Division, Department of Pediatrics-Infectious Diseases Lab, University of Oklahoma Health Science Center, Oklahoma Children’s Hospital at OU Health, Oklahoma City, OK 73104, USA

**Keywords:** breathomics, real-time breath analysis, breath volatilome, volatile organic compounds, metabolite biosynthesis, oxidative stress pathways, proton transfer reaction mass spectrometry, wearable biosensors, machine learning, clinical validation

## Abstract

Diagnostic latency limits time-sensitive care and early detection, and exhaled breath provides a rapid, repeatable window into metabolic and inflammatory chemistry. We review real-time breath sampling and analytical technologies and evaluate their readiness for clinical adoption, with emphasis on molecular pathways reflected in the breath volatilome and in exhaled breath condensate. Real-time mass spectrometry enables kinetic VOC profiling and targeted quantification, while humidity-aware sensors and wearable condensate platforms extend monitoring beyond the laboratory. Pathway-anchored interpretation links breath readouts to ketone handling, isoprenoid metabolism, nitric oxide signaling, lipid peroxidation, uremic nitrogen handling, and microbiome–host co-metabolism, but performance remains vulnerable to confounding, drift, and non-representative comparators. Translation requires standardized breath fraction control, traceable features, robust quality systems, and governed device algorithm stacks so that breath outputs inform decisions and outcomes.

## 1. Introduction

In clinically urgent conditions, deterioration may unfold more rapidly than routine testing can assess. A patient with heart failure may worsen over the course of a few hours, while laboratory measurements are checked only once daily. Respiratory infections can also progress rapidly during an emergency visit, forcing treatment decisions before confirmatory results are available. At the other end of the spectrum, early malignant disease develops slowly, yet screening is often spaced far enough apart that subtle early changes pass unnoticed. The problem is more than practical; it reflects a mismatch between the pace of biology and the pace of measurement. The consequences are familiar: delayed treatment, unnecessary hospitalization, and missed opportunities for early intervention. The issue also has a clear equity dimension. When diagnosis depends on centralized laboratories, stable supply chains, and specialized staff, the burden of delay falls most heavily on settings with the fewest resources. Under these conditions, a delayed result is not simply a technical inconvenience; it can shape the clinical outcome. For this reason, recent global health initiatives have placed increasing emphasis not only on whether a diagnostic test exists, but also on whether it can be performed at the time and place where the treatment decision is most critical [1].

Breath analysis offers a different kind of measurement because breath can be repeatedly sampled with minimal burden to the patient [2,3,4]. This analysis detects subtle physiological change that can be followed as a trajectory rather than inferred from isolated snapshots. The rationale for this approach is both biological and practical. Volatile organic compounds (VOCs) and small inorganic gases reflect systemic metabolism through blood-to-alveolar exchange, while airway-derived chemistry and the nonvolatile constituents recovered in exhaled breath condensate (EBC) reflect epithelial inflammation, oxidative stress, and processes at the airway surface. In this sense, breath analysis has the potential to narrow the gap between intermittent laboratory testing and continuous monitoring. From a molecular perspective, the breath matrix spans small inorganic gases such as CO_2_, NO, CO, and NH_3_, as well as a broad set of volatile organic compounds whose structures map to core biochemical pathways. Acetone reports ketone body metabolism, isoprene reflects flux through the mevalonate pathway, and diverse carbonyls, sulfur-containing volatiles, and aromatic species can accompany oxidative stress, airway inflammation, microbiome activity, xenobiotic biotransformation, and environmental exposure. Because the same molecular classes can arise from multiple sources, clinically useful breath signatures typically rely on multicomponent patterns and their time structure rather than a single molecule [5].

Early limitations in the breath analysis field were related to instrumentation and other factors. Breath is a chemically crowded matrix, spanning a wide concentration range and shaped by diet, medication, microbiota, and ambient exposure [5,6]. Humidity, the portion of exhaled breath sampled (for example, mixed expired air versus end-tidal air), and residual effects from the sampling inlet or tubing due to prior samples or ambient exposure can all alter the measured signal. Many early discovery studies relied on case–control separation strategies that proved difficult to sustain once real clinical heterogeneity was introduced. In practice, comorbidity and background exposure are not nuisance factors that can simply be removed. They form part of the setting in which any useful diagnostic test must work. What has improved in recent years is the maturity of the analytical platforms and the supporting evidence base. Real-time methods can now measure breath chemistry on physiologically relevant timescales. Sampling can be stabilized by engineered interfaces, and modern inference pipelines can convert high-dimensional chemical data into outputs that fit more naturally into clinical workflows [7,8]. As a result, the field has moved away from presenting breath as a direct substitute for blood tests, swabs, or imaging. Its more credible role is as a repeated temporal layer of measurement, capable of tracking transitions, treatment responses, and early deviations that more intermittent testing may miss [9].

The shift from episodic testing to repeated or near-continuous measurement brings additional demands that are not captured by analytical performance alone. A wearable device or a routine breath-sampling system does not simply generate a chemical readout. Over time, it may also produce a detailed record of physiology and health behavior. Depending on how such data are handled, they may reveal aspects of a person’s environment, daily habits, or future disease risk. For that reason, privacy protection, subgroup performance reporting, and data governance need to be built into validation from the outset rather than treated as issues to be addressed later. Recent international guidance on the ethics and governance of artificial intelligence (AI) in health points in the same direction [10]. This review is therefore organized around the question of how breath diagnostics can be used in practice rather than around instrumentation alone. We first examine the biological origins of breath signals and the main sources of noise and variability. We then consider the principal platform families, from reference-grade mass spectrometry to wearable sensors, and discuss validation pathways in relation to real clinical decisions and outcomes. Throughout, the emphasis is on the conditions under which breath measurements can be relied upon in routine care, particularly when sampling is repeated, low-burden, and performed outside a conventional clinical laboratory.

## 2. The Breath Metabolome and Volatilome: Molecular Sources, Compartments, and Confounding

**Scheme 1:**
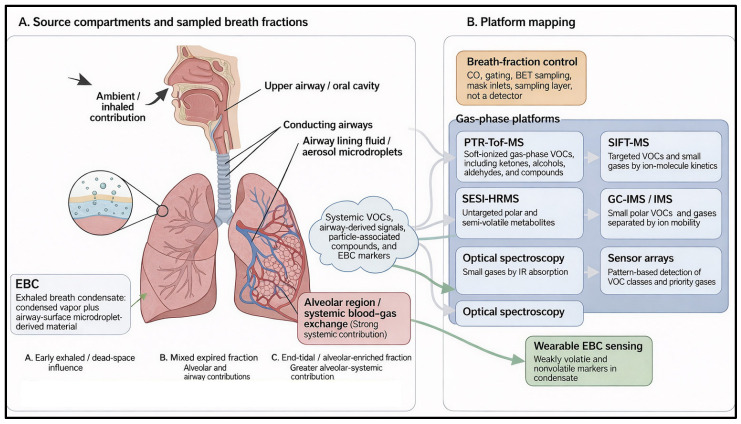
Biological sources of breath signals and corresponding measurement platforms. The scheme summarizes the main source compartments contributing to exhaled breath, the sampled breath fractions, and the principal platform classes used to measure gas-phase VOCs, small inorganic gases, particle-associated compounds, and exhaled breath condensate (EBC) markers. Breath fraction control is shown as a sampling layer rather than a detector.

**Scheme 2:**
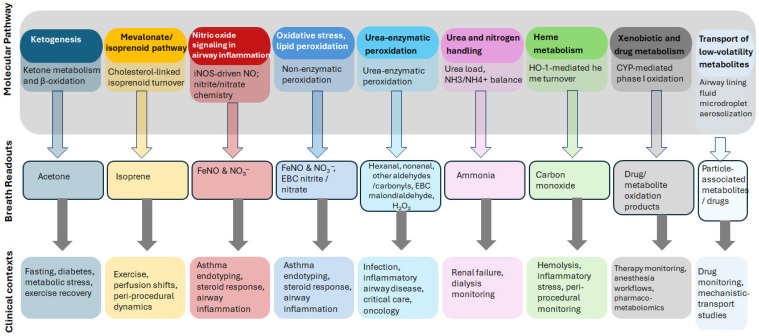
Representative molecular pathways contributing to exhaled breath signatures, linking key biochemical processes to measurable breath readouts and relevant clinical contexts.

VOCs from exhaled breath are more numerous and diverse than those from blood or urine [11]. Breath provides a composite chemical signal that is assembled from several biological and environmental sources that converge at the mouth or nose during exhalation. Some VOCs are derived from chemical reactions that have been exchanged from blood into alveolar air. Other VOCs are produced locally in the airways by epithelial metabolism, immune activity, oxidative reactions, and microbial processes. In addition, environmental VOCs are inhaled or ingested compounds, which can enter the breath directly or after partial metabolism. Exhalation also carries particles and microdroplets generated by the airway lining fluid. When these are condensed into exhaled breath condensate (EBC), the specimen becomes an aqueous matrix that contains ions, small metabolites, redox-active species, lipids, proteins, and nucleic acids. All of these can provide clinically useful information, but each is also open to dilution, contamination, and variability in breathing patterns and sampling geometry. The main contributing compartments and their corresponding content are shown in Scheme 1. To make the molecular origin of these breath signals explicit, Scheme 2 summarizes representative biochemical pathways and processes that generate common breath readouts and indicates the clinical contexts in which they are most often interpreted.

### 2.1. Systemic Blood-to-Alveolar Exchange and Partitioning Physiology

Circulating metabolites enter exhaled breath through the blood to the alveolar exchange and by partitioning into the airway lining fluid. Whether a compound is detectable in breath is governed by its volatility, solubility in blood and airway surface liquid, blood–gas partition coefficient, ventilation–perfusion matching, and the fraction of breath sampled. Compounds with low blood solubility can equilibrate rapidly with alveolar air and often track to the end tidal plateau, whereas more water-soluble species can be buffered by airway liquid and become sensitive to breathing patterns and recent wash-in and wash-out history. Regional recruitment and ventilation perfusion heterogeneity further shape the sampled fraction, so breath concentration reflects production, distribution, and exchange rather than circulating abundance alone [9,12,13].

This exchange link also supports a molecular reading of several canonical breath VOCs. Breath acetone is closely tied to hepatic ketone chemistry. During increased fatty acid beta-oxidation, acetyl-CoA accumulates and is routed into ketogenesis. HMG CoA synthase and HMG CoA lyase generate acetoacetate, and its decarboxylation produces acetone that partitions into blood and can be cleared through the lungs. Isoprene is commonly linked to the mevalonate pathway that supplies cholesterol and other isoprenoids, and it is often interpreted as arising from turnover of isoprenoid intermediates such as dimethylallyl diphosphate. Because exchange depends on perfusion and ventilation, isoprene and related small hydrocarbons can vary with circulation and respiratory dynamics as well as with biosynthesis. The circulating metabolite pool is also shaped by the gut microbiome. Microbial end products and microbially modified metabolites can enter the circulation and later appear in exhaled breath. Part of the breath signal, therefore, reflects host–microbial co-metabolism rather than host metabolism alone, especially for pathways linked to fermentation, sulfur turnover, nitrogen handling, and aromatic compound degradation. Clinical and dietary studies have shown strong associations between breath volatiles and gut microbiome composition and function, and dietary intervention can shift breath VOC profiles in reproducible ways [14,15]. More recent mechanistic work has strengthened this view by showing that the microbiota shapes the breath volatilome across species [16]. The measured spectrum is also conditioned by the analytical front end. Humid breath promotes ion clustering and surface losses, and inlet memory can selectively distort reactive carbonyls and sulfur species unless materials and calibration are selected for humid matrices [9,12,13].

### 2.2. Airway Surface Chemistry and Exhaled Breath Condensate

Airway-derived signals represent a biological layer distinct from systemic exchange. The chemically active airway surface contains epithelial cells, immune cells, mucus, and resident microbes that reshape local redox balance and nitrogen chemistry. A well-defined example is exhaled nitric oxide. In inflammatory airway disease, cytokine-driven induction of inducible nitric oxide synthase in airway epithelium increases conversion of L-arginine to nitric oxide and L-citrulline, thereby raising FeNO during eosinophilic inflammation and altering its response to corticosteroid therapy [17,18]. Nitric oxide is also oxidized to nitrite and nitrate, and these anions can be recovered in exhaled breath condensate, linking a gas-phase signal to an aqueous proxy of airway lining fluid. Oxidative stress adds a second mechanistic axis. Reactive oxygen species generated by activated inflammatory cells and stressed epithelium can initiate lipid peroxidation in membrane polyunsaturated fatty acids. Breakdown of lipid hydroperoxides yields aldehydes and related carbonyl products, including malondialdehyde and 4-hydroxy-2-nonenal, alongside smaller aldehydes that are sufficiently volatile to appear in the gas phase. Because many of these products are reactive and short-lived, their detectability depends on the sampled fraction, the timing of collection, and the extent to which inlet chemistry and humidity alter recovery.

Exhaled breath condensate expands the accessible chemistry beyond VOCs by condensing microdroplets from airway lining fluid into an aqueous matrix. The matrix can contain ions, metabolites, redox markers, proteins, nucleic acids, and other airway surface constituents, but its translational value depends on rigorous control of dilution, oral carryover, and analyte stability. These challenges were formalized early and remain central to interpretation and cross-study comparability [19,20,21]. Methodological frameworks and technical standards emphasized the need to specify the collection device, temperature control, breathing maneuver, and normalization strategy, since device-dependent recovery and saliva contamination can dominate apparent biological differences if they are not measured and corrected [13,22,23]. EBC is therefore best positioned as a protocol-sensitive but high-yield matrix for longitudinal monitoring and phenotype stratification, where within-person trajectories can be interpreted under consistent collection conditions.

### 2.3. Particle Phase Transport of Low Volatility Metabolites and Nonvolatile Compounds

Breath is often discussed as a volatilome, but exhalation also carries suspended particles and microdroplets generated from airway lining fluid. These particles form when small airways and alveolar regions undergo transient closure and reopening, which creates thin liquid films that can rupture and aerosolize. The chemistry of these droplets is not random. Airway lining fluid is enriched in pulmonary surfactants, a lipoprotein-like mixture composed primarily of phospholipids and cholesterol, with associated surfactant proteins. Because surfactant components are amphiphilic, they stabilize the air–liquid interface and can also solubilize and concentrate hydrophobic metabolites, oxidized lipids, and drug molecules within a lipid-rich microenvironment. In parallel, the aqueous fraction of droplets carries dissolved salts, small polar metabolites, and peptides. This creates a transport route in which molecules with low intrinsic volatility can still appear in breath because they are exported in the particle phase rather than transferred via blood–gas equilibrium. Chen et al. [24] demonstrated this mechanism with unusual clarity by tracking venlafaxine after systemic administration in mice. Despite being predominantly ionized and nonvolatile, venlafaxine was detected in exhaled breath in association with particles, and its time course followed the blood pharmacokinetic profile. This observation matters beyond a single drug example. It shows that the boundary between gas-phase VOC analysis and airway fluid-adjacent sampling is less strict than it appears, and that some circulating compounds can reach the exhaled stream through aerosolization of airway surface material.

Particle phase readouts also introduce a distinct confounder class. Particle number and size distribution depend on breathing mechanics, airway hydration, and the closure and reopening behavior of small airways, all of which can shift with disease state and with the sampling maneuver itself. For that reason, particle-associated biomarkers should be interpreted together with respiratory pattern metadata and, where feasible, direct particle metrics. Without this context, changes in droplet generation or sampling geometry can be misread as biochemical change, particularly for analytes that preferentially partition into surfactant-rich droplets.

### 2.4. Molecular Confounders and the Value of Dynamic Measurements

Breath is chemically informative because it is produced at the boundary between internal metabolism and the external environment. The same property makes confounding difficult to avoid. Volatile organic compounds present in room air can be inhaled and later reappear in exhaled breath, producing a wash-in and wash-out behavior that can resemble endogenous generation. This is particularly relevant for common indoor volatiles and solvents, where the breath signal can be shaped by recent exposure history as much as by physiology. Diet and oral chemistry add another strong layer of variation. Ingestion studies show that everyday exposures can introduce dozens of reproducible features into the breath profile within minutes, even when those features are not related to disease biology unless the exposure itself is measured and accounted for [25]. Oral processes can also reshape breath composition through rapid chemical and microbial transformations at the mouth and upper airway surface, which can dominate parts of the spectrum if sampling timing and breath fraction are not controlled.

Smoking adds confounding through two mechanisms that are hard to separate in routine cohorts. First, cigarette smoke contributes direct exogenous volatiles and reactive carbonyls that can persist in the airway and sampling pathway. Second, chronic smoking alters airway biology through sustained inflammation and structural remodeling, which changes endogenous breath chemistry even after recent smoking is excluded. Variation is also present in healthy individuals. Stable person-specific breath profiles and time-of-day effects can explain a substantial share of the measured variance and may obscure early disease-related changes when cohorts are sampled under different schedules or routines [26]. These observations are a reminder that confounding is not an occasional nuisance in breath studies. It is a baseline condition that must be measured and modeled. Microbiome-related variation should be treated in the same way. It is not simply background noise, because it can reflect the genuine host and host microbial biology [14,15,16]. A clear molecular example is the production of short-chain fatty acids by gut bacteria. Acetate, propionate, and butyrate arise from fermentation of dietary substrates through conserved bacterial pathways, including acetate formation from acetyl CoA, propionate formation through succinate or propanediol routes, and butyrate formation through butyryl CoA intermediates. Underlying mechanisms are encoded at the gene level in the microbiome through pathway-specific enzymes, and differences in pathway abundance can shift the circulating pool of these metabolites and related derivatives that may be detected in breath under appropriate analytical conditions. In practical terms, this means that diet-driven shifts in microbiome metabolism can create structured and repeatable breath changes that are biologically real, yet clinically misleading if the diagnostic question is unrelated to nutrition or gut microbial activity.

For translation, the key question is whether a model remains reliable when this degree of real-world variability is present [6]. Repeated measurement offers one defensible way to address the problem. Within-person trajectories can be more stable and more informative than population cut-offs, particularly when the goal is early warning, treatment adjustment, or recovery monitoring. This aligns with the growing interest in VOC dynamics, where kinetic information helps distinguish endogenous production from recent exposure and can reveal responses to intervention that are missed by single-time point sampling [9]. At the same time, disease specificity is rarely achieved at the level of one molecule, because many breath signals converge on shared biochemical processes such as oxidative stress, lipid peroxidation, and microbiome-linked metabolism. What is most likely to prove useful in practice is a multivariate biochemical pattern that remains interpretable in the presence of confounding, reproduces across sites, and is stable enough to support clinical action. Seen in this way, the analytical platform is not only a technical choice. It determines which part of chemical space is captured and whether that information can be stabilized sufficiently for routine clinical use.

### 2.5. Molecular Classes and Biochemical Origins of Breath Analytes

From a molecular perspective, breath biomarkers are best interpreted as chemical products of a limited set of biological processes rather than as disease labels. Most candidates fall into recurring chemical families, and each family points to a plausible origin in intermediary metabolism, redox chemistry, microbial transformation, or xenobiotic handling. This framing helps explain why single-molecule claims often fail outside curated cohorts, while multicomponent patterns and kinetics can remain informative under realistic heterogeneity [9,11]. Ketones, such as acetone, and related alcohols formed via reversible redox chemistry can signal shifts in hepatic energy balance and systemic NADH availability. Isoprene is an abundant endogenous hydrocarbon that is linked to isoprenoid biosynthesis and can vary with metabolic state and ventilation. Carbonyls, including aldehydes and ketones, often increase with oxidative and inflammatory stress because lipid peroxidation and secondary oxidation pathways generate volatile fragments, a mechanism that has motivated real-time monitoring of simple hydrocarbons in acute settings [27]. Nitrogen-containing species span nitric oxide and its downstream chemistry in the airway, as well as ammonia linked to urea handling and microbial urease activity. Sulfur-containing volatiles reflect methionine and cysteine turnover and microbial metabolism, and they can be strongly shaped by oral and airway microbiota. Many high-value airway markers are not volatile. Exhaled breath condensate provides access to ions and redox-active molecules that are difficult to measure in gas-phase panels, including nitrite, nitrate, ammonium, hydrogen peroxide, lipid peroxidation products, and selected proteins. The translational challenge is that these species are present in a dilute, protocol-sensitive matrix, so collection efficiency, normalization strategy, and stability controls become part of the molecular assay definition [17]. These molecular origins map naturally onto the technology stack summarized in Scheme 1. Proton transfer and selected ion flow tube mass spectrometry emphasize oxygenated VOCs and other proton-affinity-driven volatiles; optical spectroscopy anchors small inorganic gases through calibrated absorption physics; and ambient ionization, high-resolution workflows extend coverage toward polar and semi-volatile metabolites. Sensor arrays and wearable condensate systems can then be tuned toward priority chemical families, if humidity, drift, and quality control are engineered into the measurement chain.

## 3. Real-Time Platforms for Breath Molecular Measurements

**Figure 1:**
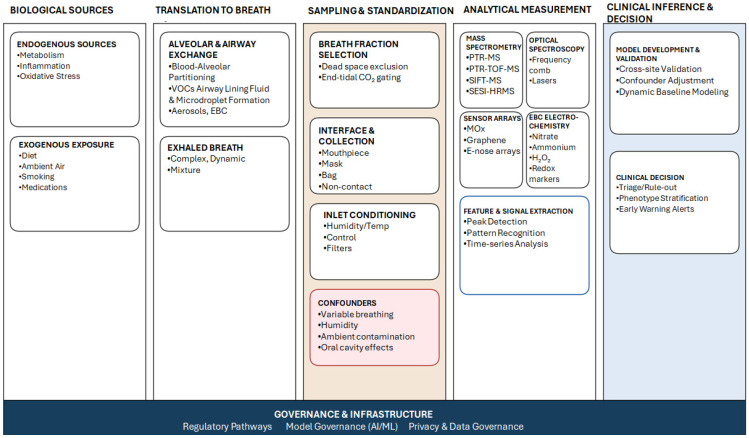
The journey of a breath biomarker from source to clinical decision. Schematic traces endogenous production and exogenous exposure through blood-to-alveolar exchange and airway-surface chemistry, followed by sampling fractionation, inlet conditioning, analytical measurement, feature extraction, and clinical inference layers. It emphasizes where confounders enter, where standardization acts, and where dynamic monitoring converts variability into interpretable trajectories.

**Figure 2:**
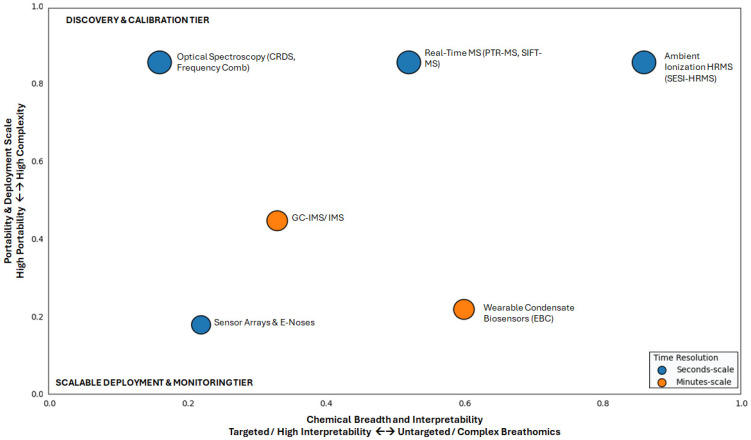
Technological ecosystem of real-time breath analysis. Platforms are positioned by chemical breadth and interpretability on the *x*-axis and portability and deployment scale on the *y*-axis. The landscape separates into a discovery and calibration tier and a scalable monitoring tier, highlighting the need for hybrid workflows that link reference platforms with portable devices. Abbreviations: CRDS, cavity ring-down spectroscopy; EBC, exhaled breath condensate; GC-IMS, gas chromatography–ion mobility spectrometry; HRMS, high-resolution mass spectrometry; MS, mass spectrometry; PTR, proton transfer reaction; SESI, secondary electrospray ionization; SIFT, selected ion flow tube.

**Figure 3:**
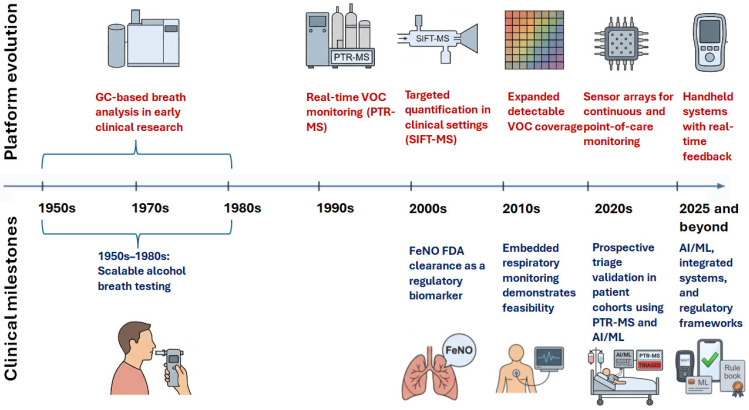
Evolution of breath-analysis platforms and milestones toward clinical adoption. The upper timeline summarizes major developments in analytical platforms, from early gas chromatography to real-time mass spectrometry, targeted ion–molecule methods, broader VOC coverage, sensor arrays, and emerging handheld systems. The lower timeline highlights corresponding milestones in clinical uptake, including alcohol breath testing, FeNO clearance, embedded respiratory monitoring, prospective triage validation, and the current move toward AI-assisted, workflow-integrated, and regulation-aware breath diagnostics.

Real-time breath analysis is not built around a single technology. It consists of a group of analytical approaches, each with its own balance of chemical coverage, speed, cost, portability, and ease of interpretation. In practice, this has led to a layered technical structure. Reference-grade instruments are most useful at the discovery stage, where the aim is to identify candidate markers, assign chemistry where possible, and monitor calibration and drift. Smaller or less expensive systems become more relevant once the question shifts toward routine monitoring, where rapid turnaround, simple operation, and minimal disruption to workflow are often more important than complete molecular attribution. Benchmarking studies provide an important point of comparison across these platforms. Standardized exposure and washout protocols, such as peppermint challenge designs, are particularly useful because they can be repeated across instruments and across sites [28,29,30]. Figure 1, Figure 2 and Figure 3 summarize the overall translational pathway, the current technological landscape, and the main stages in the development of breath-analysis platforms. Table 1 summarizes recurrent breath analytes and analyte classes together with their likely origin, main clinical context, suitable platform types, and the main factors that complicate interpretation.

### 3.1. Real-Time Mass Spectrometry: Broad Chemical Coverage with Fast Kinetics

PTR-MS and PTR-ToF-MS enable tracking of breath chemistry on the timescale of physiological changes [30,31]. Their analytical advantage is soft chemical ionization with limited fragmentation, which enables continuous monitoring of chemically distinct VOC families during rest, exercise, feeding, and acute illness [32]. The measured spectrum includes abundant low-molecular-weight metabolites and lower-abundance compounds linked to oxidative stress, inflammation, and host–microbial co-metabolism. Representative pathway-linked examples are summarized in Table 1. Acetone is often interpreted in relation to beta oxidation and ketone handling, whereas isoprene is associated with mevalonate-derived isoprenoid turnover. Aldehydes and related carbonyls can increase during lipid peroxidation, and sulfur volatiles and short-chain fatty acids are frequently shaped by diet and airway or gut microbial activity. In most settings, these signals are most useful as readouts of pathway activity rather than as disease-specific markers. PTR-based instruments, therefore, sample a mixture whose components differ in origin, transport behavior, and time course. In perturbation studies, kinetics often carry more information than a single concentration snapshot. Many VOCs change over minutes, so features such as onset, peak timing, and washout can reflect production, distribution, and clearance more directly than an isolated measurement [33]. Repeatability improved once sampling shifted from poorly defined exhaled air to controlled breath fractions. Buffered end-tidal sampling reduces dead-space variation, while higher-resolution time-of-flight designs reduce mass overlap and improve feature separation, allowing more consistent detection across sessions [12,34,35]. These engineering steps help to ensure that measured variation reflects biology rather than differences in breathing maneuvers or collection methods. Prospective respiratory cohorts further show that PTR-ToF-MS breath prints can retain useful performance under heterogeneous clinical conditions, particularly when evaluation uses realistic differentials rather than healthy controls alone [8,36]. A persistent limitation of real-time MS is quantitative integrity for reactive and surface-active VOC classes.

Some of the compounds most informative about inflammation and oxidative stress are also the hardest to measure accurately. Aldehydes, certain sulfur-containing species, and other reactive VOCs often carry the most mechanistically relevant information, but they are also particularly sensitive to inlet memory, humidity, and surface losses. If the front end is not carefully designed and calibrated under humid breath-like conditions, the profile may shift toward more stable surrogate compounds that are easier to detect but less closely linked to the underlying biology [37]. This is one reason layered analytical strategies are often preferred when pathway traceability is important [38,39]. In such workflows, real-time mass spectrometry provides kinetics and throughput, while a smaller group of higher-value features is checked periodically by an orthogonal method. The confirmation of short-chain fatty acids by TD-GC-MS provides a good example of this approach [40]. SIFT-MS complements PTR-based methods by offering direct quantification based on established ion–molecule reaction kinetics [41,42]. A practical advantage is that it can be used with clinical gas lines and ventilator circuits, which makes breath monitoring easier to incorporate into routine patient care rather than treating it as a separate procedure [43]. At the discovery end, ambient-ionization high-resolution platforms such as SESI-HRMS extend coverage toward more polar and semi-volatile metabolites [44]. The limitation is that the data are highly context-dependent. Ingestion effects and diurnal variation can easily dominate the signal unless they are directly measured and accounted for [25,26]. In asthma, some of the most convincing HRMS studies have therefore been those built around intervention, where breath chemistry was used to distinguish response phenotypes and metabotypes rather than to carry the greater burden of cross-sectional disease classification [45]. Real-time mass spectrometry remains the primary reference platform in breath diagnostics because it can identify promising breath markers, detect key confounding factors early, and track signal changes over time. That information can then be used to build smaller, simpler systems for routine clinical use, including compact separation platforms and sensor-based devices.

### 3.2. Ion Mobility Platforms: Portable Detection with Constrained Attribution

Ion mobility spectrometry, including GC-IMS, has found a practical niche in breath analysis. Compared with full mass spectrometry, these systems are smaller, run faster, and are easier to operate, which is why they remain attractive for point-of-care use and for repeated measurements in clinic-based studies. These systems are useful because they separate ionized breath compounds according to how quickly they move through gas. Rather than trying to identify many compounds simultaneously, they simplify a complex breath mixture into a smaller set of measurable signals. For focused clinical applications, such as ammonia monitoring in renal disease, that level of information is often sufficient. However, handling humidity is a technical challenge. Breath is a wet matrix, and water alters the ion population within the IMS cell by influencing clustering and the reactant–ion balance. Peak position and peak intensity can therefore drift even when the underlying breath composition has not changed. This is one example of why humidity cannot be treated as a minor operating variable. Ion mobility spectrometry (IMS) and gas chromatography coupled to IMS (GC-IMS) sit in the deployment-friendly zone because they can be compact, relatively fast, and less demanding to operate than full mass spectrometry. Their main limitation is that the mobility signature depends strongly on matrix conditions, which are difficult to maintain constant during routine breath sampling. Water vapor shifts the reactant ion balance and ion clustering, which can move peak position and intensity and reduce the reliability of library matching and multivariate feature spaces unless humidity is measured and actively managed. This matters in practice because exhaled breath is saturated with water, and deviations from the calibration envelope can translate into drift in peak position or quantitative recovery. Deployable IMS workflows therefore need an engineered humidity strategy, combined with routine quality control checks that flag when spectra have moved outside the calibration domain. These platforms are most reliable when the analyte space is deliberately constrained, and the sampling envelope can be standardized, for example, in dialysis-associated ammonia monitoring or in structured screening sessions [46].

### 3.3. Optical Spectroscopy: Precise Quantification for Selected Analytes

Optical spectroscopy has generally been developed as a targeted and metrologic approach to breath analysis rather than as a discovery platform [47,48]. Photoacoustic implementations have been described in parallel for a range of selected analytes [49,50]. Its main advantage lies in the mid-infrared region, where many breath constituents exhibit strong, highly specific absorption bands. Under these conditions, concentration can be directly derived from light absorption, with less ambiguity than is typically encountered in pattern-based methods. Frequency-comb and other cavity-enhanced systems can detect multiple gases in real-time with high sensitivity, making them particularly suitable for applications that require both specificity and calibration stability. This is especially relevant for continuous measurements of CO_2_, CO, NO, ammonia, and selected small VOCs with strong infrared signatures [7]. In this role, optical methods are used less to search broadly for new biomarkers and more to provide traceable measurements of compounds whose physiological interpretation is already reasonably well established. A further strength of optical methods is their ability to resolve isotopologues. This extends breath analysis from measuring concentration alone to examining origin and turnover. Real-time measurements of ^13^C- and ^18^O-labeled CO_2_ with per-mil precision have already been demonstrated at minute-scale integration, with stable tracking across the alveolar plateau and good preservation of isotope fidelity [51]. This type of measurement is well-suited to tracer studies and metabolic phenotyping, and it may also serve as an external reference for validating or recalibrating broader breathomics workflows. The limitations of optical spectroscopy arise from the same feature that gives it strength. These systems only detect compounds that are addressed by the chosen wavelengths. They do not provide broad chemical coverage by default. In addition, water vapor and CO_2_ dominate substantial parts of the infrared spectrum, so routine breath measurements often depend on careful control of humidity, temperature, and optical-path stability. For these reasons, optical spectroscopy is best regarded as a reference method for targeted biomarkers and physiology-based tests, and as a useful calibration layer within broader breath diagnostic systems, rather than as a stand-alone solution for open-ended VOC discovery. Nevertheless, photoacoustic approaches are beginning to show disease-specific potential in obstructive airway disease [52].

### 3.4. Sensor Arrays and Contactless Transducers

Sensor-based approaches are appealing for breath analysis because they can be small, inexpensive, and low-power, features that make repeated sampling outside specialist laboratories more realistic. The difficulty lies in the sample itself. Exhaled breath is warm, saturated with water, and chemically complex, while most sensor materials respond to classes of compounds rather than to a single analyte. As a result, the output is usually a composite pattern influenced by chemistry, humidity, flow, and the sensor’s recent history. Progress in this area has come less from the discovery of one ideal sensing material than from treating the device as a full measurement system, including the sensing layer, the package, the calibration strategy, and the method used to interpret the signal. This is illustrated by the development of array-based systems with scalable electronics. Complementary metal-oxide semiconductor (CMOS)-integrated graphene and metal-oxide arrays have demonstrated rapid responses, along with practical approaches to drift management and interference correction, both of which are essential if such systems are to move beyond proof-of-concept studies [53]. Terbium-doped SnO_2_ yolk–shell chemiresistors were designed to reduce the effect of humidity while preserving sensitivity to targets such as acetone, which remains of interest in metabolic monitoring [54]. Device format matters as well. A Pd-WO_3_ acetone sensor incorporated into a disposable mask provides a good example of how chemical sensing can be adapted for repeated use in a simple, acceptable form for the wearer [55]. Other transduction approaches widen the range of possible designs when low-burden operation is the main goal. A magnetoelastic contactless sensor, for example, has been used to distinguish breath from ambient air while simultaneously tracking humidity and selected gases, suggesting a simpler route for wearable monitoring when exact molecular identification is not essential [56]. Flexible ammonia sensors based on tungsten-doped vanadium dioxide and carbon composites provide a related example, particularly for renal and environmental monitoring, where a wearable format may be advantageous [57]. It is also worth noting that breath pattern sensing is not merely an accessory to chemical detection. In many cases, it provides the context needed to interpret chemistry. QCM-based humidity sensors, smart textiles, and optical-fiber systems can all detect breathing patterns and pauses over short time scales [58,59,60]. They may therefore help define when and under what conditions a signal was generated. These developments suggest a practical role for sensor-based systems. They are unlikely to replace platforms that offer clear molecular attribution, but they become much more viable when humidity and drift are handled explicitly and when the chemical signal is interpreted alongside breathing metadata and calibration procedures that help preserve stable inference across users and environments.

### 3.5. Wearable EBC Sensing

Mask-based condensers combined with simple microfluidic handling are beginning to change how exhaled breath condensate is collected [61]. Instead of being limited to slow, supervised sampling in research settings, EBC can increasingly be obtained repeatedly during routine activity. This is important because EBC is more than condensed breath water. It contains airway-lining fluid microdroplets and dissolved compounds, thereby providing access to ions and other low-volatility markers that are only weakly represented in gas-phase VOC profiles. In practical terms, EBC extends breath analysis toward airway surface chemistry, including markers of inflammation and oxidative stress that are often better suited to longitudinal monitoring than to one-off diagnosis. The main obstacles at this stage are less about whether such biomarkers exist and more about how they are collected and measured. Condensate volume is influenced by breathing patterns, humidity, and cooling efficiency, so dilution and normalization need to be addressed explicitly if changes in collection are not to be mistaken for changes in biology. Oral carryover and salivary contamination remain persistent concerns, particularly for analytes that are abundant in the mouth. Temperature control is also important, since some targets may degrade, volatilize, or shift equilibrium during collection and storage. Electrochemical approaches are attractive in this setting because they enable the direct measurement of selected redox and lipid peroxidation markers in a humid matrix [62,63]. Their clinical usefulness, however, will depend on whether the full platform can provide stable within-person trajectories, supported by defensible normalization and built-in quality checks, rather than isolated measurements that are difficult to compare from one session to the next.

### 3.6. Platform Comparison and Translational Readiness

When these platforms are considered side by side, the main translational issue is not which performs best in abstract analytical terms, but which can produce a result that remains stable, interpretable, and useful in the setting in which it is intended to be used. In practice, the different platform families make different compromises. Some offer broader chemical coverage, others offer greater portability, faster turnaround, or simpler calibration. For this reason, platform comparison is less a matter of ranking technologies than of asking which approach is best suited to a particular clinical purpose. As the field develops, the emphasis is beginning to shift away from analytical performance considered in isolation and toward clinical usefulness. In most situations, the goal is not to catalog every detectable molecule in breath, but to obtain a readout that can support a defined clinical action. Large COVID-19 studies have been valuable in this respect because they tested workflow integration and performance under heterogeneous conditions. At the same time, they do not show that the field has reached the same level of maturity across other diseases or across routine seasonal settings. The next stage will therefore depend on broader prospective studies across a wider range of indications, together with benchmarking strategies that are not tied to a single disease wave or sampling context. Standardized exposure and washout protocols are useful in this regard, since they can be repeated across centers and used to examine the robustness of sampling, calibration, and inference. Table 2 compares the major real-time breath platform families based on analytical scope, translational constraints, and likely clinical role.

## 4. Clinical Translation: From Molecular Signature to Diagnosis

In breath diagnostics, the main challenge is usually not detecting a chemical signal but interpreting that signal in a real clinical setting. A breath profile may be statistically distinct and still have limited practical value if it is not linked to an actionable clinical insight. For this reason, the intended use of breath diagnostics in translational studies needs to be defined at the outset, whether the aim is screening, triage and rule-out, treatment adjustment, early warning, or surveillance after therapy. The intended use then defines the choice of comparator groups, and the study can be judged in relation to the consequences of false-positive and false-negative results within the test’s workflow. Many breath signals reflect processes that are biologically important but not unique to a single disease, including airway inflammation, oxidative stress, microbiome-related change, and broader shifts in systemic metabolism. Such signals may still be clinically useful, but they do not necessarily provide specificity on their own. This is particularly relevant in higher-stakes applications such as oncology, where breath is likely to be most useful when interpreted alongside other clinical data. In practice, this means combining breath features with routine clinical variables, physiologic measurements, or imaging findings so that the result aligns more closely with how decisions are already made in the clinic. Table 3 summarizes representative validation studies in which sampling strategy, cohort heterogeneity, and evaluation design were aligned with defined clinical use.

### 4.1. Infectious Disease Triage and Syndromic Screening

Infectious disease triage is one setting in which delay in diagnosis matters most. Decisions on isolation, bed placement, treatment, and escalation of care are often taken before laboratory confirmation is available. Breath sampling is attractive in this context because it is quick, non-invasive, repeatable, and does not compete with the same laboratory capacity as swabs or blood tests. The biological rationale is that acute infection, along with the host response, can alter the levels and patterns of VOCs and small gases [66]. At the molecular level, infection reshapes breath chemistry through two coupled sources. The first is host immunometabolism. Pattern recognition signaling rapidly shifts substrate use and redox balance, and activated neutrophils and macrophages generate reactive oxygen and nitrogen species. These oxidants initiate lipid peroxidation in membranes and lipoproteins, yielding volatile carbonyls such as aldehydes that can rise alongside inflammation. The second source is microbial metabolism and microbe-driven remodeling of the airway surface, which can introduce nitrogen and sulfur-containing volatiles and alter the balance of small organic acids. These mechanisms help explain why breath signals can change within hours, but they also clarify the specificity problem in syndromic illness. Many of the same oxidative and inflammatory pathways are shared by viral, bacterial, asthma exacerbation, and chronic lung disease flare. This is one reason why breath performs best as an early probability signal that accelerates decisions, rather than as a standalone molecular diagnosis [66]. Breath is therefore being explored not as a replacement for nucleic acid testing, but as a way to provide an early, rapid signal in diagnosis and treatment. The studies that have shown the most promise have generally used breath as a front-end screening tool. In that role, the result can contribute to rapid rule-out, help identify who should proceed to confirmatory testing, and support prioritization when the pre-test probability is uncertain. Liangou et al. [36] reported that PTR-ToF-MS models maintained useful screening performance across a large multisite dataset despite substantial clinical heterogeneity, although the stronger performance observed in older subgroups is probably better interpreted in relation to the study population than as a general benchmark. Roquencourt et al. [8] also showed that rapid breath analysis could be incorporated into a prospective hospital triage workflow. Breath-based approaches have also been explored in ventilator-associated lower respiratory tract infections, where an untargeted analysis demonstrated feasibility in a critical care setting [64]. At the same time, these studies point to an obvious limitation. In syndromic respiratory illness, high sensitivity is only part of the problem. Specificity is constantly challenged by common alternatives, such as influenza-like illness, bacterial pneumonia, and exacerbations of chronic lung disease, as well as by changes in prevalence from one season to the next. For this reason, breath triage is likely to be most useful when it is linked to a clear downstream action. A probabilistic result may be used to trigger confirmatory testing, to support earlier de-escalation of isolation in lower-risk patients with a negative signal, or to prompt more directed work-up when the signal is positive in a higher-risk setting. In the end, the value of breath testing in this area is better judged by what it changes in the workflow than by discrimination metrics alone.

### 4.2. Respiratory Disease, Inflammation, and Treatment Response Phenotyping

Respiratory disease is an obvious area in which breath analysis might be expected to perform well, given the proximity of the signal to the organ of interest. At the same time, it is also an area marked by substantial clinical heterogeneity [67]. Symptoms such as cough and dyspnea are shared by asthma, COPD, post-infectious syndromes, heart failure, and anxiety; thus, detection of a single compound is rarely specific enough to establish a diagnosis. For this reason, breath is more likely to be useful when it is used to describe an inflammatory state or response to treatment rather than to classify disease as simply present or absent [68,69,70]. The strongest molecular anchor in airway breath testing is nitric oxide. In type 2 inflammation, cytokine signaling upregulates inducible nitric oxide synthase in the airway epithelium, increasing nitric oxide production and raising FeNO. Because nitric oxide chemistry is directly connected to inflammatory signaling and steroid responsiveness, FeNO has become clinically interpretable when sampling is standardized [17,18]. That same pathway is also evident in condensate chemistry, as nitric oxide is converted to nitrite and nitrate in airway lining fluid, and these ions can be recovered in exhaled breath condensate under controlled collection conditions [19,20,21,22,23]. In parallel, oxidative pathways contribute additional molecular classes. Peroxide-generating reactions and lipid peroxidation can yield redox-active markers in condensate and reactive carbonyls in the gas phase. This explains why aldehydes, peroxide-related species, and malondialdehyde-type signals often recur in airway inflammation studies, even though they rarely map to one diagnosis on their own [63]. In that setting, the relevant comparison is often not between patients and healthy controls, but between different physiological states within the same individual following a defined intervention. This is where real-time breathomics may offer a practical advantage. By capturing coordinated changes across multiple metabolites, it can reflect shifts in airway metabolism, oxidative stress, and energetics. In pediatric asthma, SESI-HRMS identified hundreds of features that changed after salbutamol administration and also distinguished metabolites associated with poor bronchodilator responsiveness [45]. This intervention anchored design is molecularly attractive because it reduces reliance on cross-sectional disease labels and instead tests whether breath tracks defined biochemical shifts. Bronchodilator response changes ventilation distribution and airway caliber within minutes, and that can immediately alter the exchange kinetics of highly volatile metabolites, while anti-inflammatory treatment is expected to modify epithelial redox state and inflammatory mediator production over longer windows. When the comparison is within the same patient before and after a known perturbation, breath features can be interpreted as pathway-responsive markers rather than as static disease fingerprints [45]. This is of interest because it relates directly to a clinical question that already shapes management, namely, whether a patient is likely to respond to bronchodilator treatment and how far anti-inflammatory therapy may need to be intensified. Wearable and mask-based EBC systems extend this approach beyond the clinic by making repeated sampling more feasible in daily life, where within-person change becomes the main source of information. A smart-mask platform, for example, enabled minute-scale EBC collection and measured airway and metabolic markers that correspond with established clinical measures in cohorts with asthma, COPD, and post-COVID conditions [61]. The next question is whether such measurements can improve outcomes in practice [71]. That includes whether they can identify exacerbations before symptoms become obvious, reduce admissions through earlier intervention, or support more targeted prescribing by helping distinguish inflammatory relapse from noninflammatory dyspnea.

### 4.3. Metabolic Physiology, Exercise, and Gut-Linked Metabolites

Metabolic applications of breath analysis are moving away from single-markers and toward a more physiological reading of the breath profile as it changes over time. This is important because several of the compounds measured in breath are closely tied to metabolic state. Fortunately, the biochemistry behind the most common metabolic breath volatilecompounds is well defined. Acetone reflects ketone handling, since hepatic ketogenesis produces acetoacetate and β-hydroxybutyrate, and acetoacetate can yield acetone that partitions efficiently into alveolar air. Its breath level, therefore, reflects the integration of fatty acid oxidation rate, hepatic output, and peripheral utilization, and is expected to shift with fasting, exercise intensity, and insulin availability rather than with a single disease label. Isoprene is often linked to the mevalonate pathway and cholesterol-related isoprenoid metabolism, but in breath, it is also strongly shaped by perfusion and ventilation dynamics, which is why exercise and recovery experiments show marked time structure. Real-time platforms add molecular value here because they can track pathway-linked kinetics rather than isolated concentrations, including detection of metabolites associated with central carbon metabolism during graded exertion [72,73]. Acetone is commonly linked to fat oxidation and ketone metabolism, while isoprene has been associated with aspects of cholesterol metabolism and muscle-related physiology. Additional volatile signals change rapidly during exertion as perfusion and ventilation shift. In this setting, timing matters. A single concentration measured at a single time point may be less informative than tracking how the breath signal changes over time. This broader view is supported by studies showing that tricarboxylic-acid-related metabolites and other intermediates can be detected in exhaled breath when sampling is fast enough to follow exercise and recovery [72]. Exercise studies have also shown that multiple metabolites change with increasing intensity, consistent with a coordinated metabolic response rather than the behavior of a single biomarker [73]. One possible route toward wider use is the combination of portable sensors with models trained against physiological comparators. Breath analyzers coupled to deep learning approaches have already been used to estimate exercise-related fat burning from oral and alveolar breath features, suggesting that deployable systems may provide useful metabolic readouts when the validation strategy is sufficiently rigorous [74]. A further area of interest is the contribution of gut-derived metabolites. Gut-linked volatiles can also be described in terms of explicit pathways. Short-chain fatty acids arise from microbial fermentation of dietary substrates through acetyl-CoA and propionyl-CoA-centered routes, and a fraction of these products enters the circulation and can be detected in breath when measurement sensitivity and sampling control are sufficient [40]. The same principle applies to microbially modified aromatic compounds and sulfur-containing metabolites, which can act as readouts of host microbial co-metabolism rather than host metabolism alone [14,15,16]. This molecular framing helps with interpretation, because it makes clear why diet, fiber intake, and antibiotic exposure can shift breath profiles in reproducible ways that are biologically real but not necessarily disease specific [40]. Microbial metabolism generates VOCs that can enter the circulation and later appear in breath. Short-chain fatty acids are an obvious example, and their measurement in breath, supported by orthogonal confirmation, provides a useful analytical starting point for this line of work [40]. The main difficulty is interpretation. These compounds are often strongly influenced by diet and recent exposure. As a result, a signal may be analytically reproducible but clinically uninformative. Progress in this area is therefore likely to depend on study designs that explicitly record and model diet and exposure, rather than treating them as secondary sources of noise.

### 4.4. Peri-Procedural and Critical Care Monitoring

High acuity settings offer a direct route to adoption because the sampling interface can be stabilized by existing infrastructure such as ventilator circuits and anesthesia workflows. When flow and timing are controlled, breath readouts can be interpreted as continuous physiology rather than as isolated snapshots. SIFT-MS integrated into ventilation lines has demonstrated real-time tracking of multiple exhaled compounds in perioperative care, showing that breath measurement can be embedded into complex environments without adding behavioral burden [43]. Oxidative injury is a second mechanistically anchored target. During cardiac surgery, ischemia and reperfusion can drive lipid peroxidation and generate volatile end products such as ethylene, which has been monitored in real-time, provided that circuit effects and ambient contamination are explicitly managed [75]. Other rapid approaches underline the same monitoring-first logic by prioritizing timeliness and workflow integration over exhaustive molecular attribution [76].

### 4.5. Renal and Uremic Metabolism Monitoring

Breath ammonia is one of the more physiologically interpretable markers in breath analysis because it is closely related to urea metabolism and nitrogen balance [77,78]. In patients with renal failure, rising blood urea levels and changes in acid–base status increase the availability of ammonia and ammonium, which in turn can be reflected in exhaled breath. This has made breath ammonia an attractive candidate for serial monitoring during dialysis. In hemodialysis studies using ion mobility and optical methods, breath ammonia was higher at baseline in dialysis patients, fell during treatment, and correlated with blood urea nitrogen and adequacy measures such as Kt/V [46]. These observations are important because they link the breath signal to a physiological process that is already familiar in renal care. They also show that the marker can be measured repeatedly without burdening the patient and that the results can be related to a clear clinical question. For these reasons, renal monitoring provides a useful test case for the development of sampling interfaces, calibration procedures, and longitudinal analytical approaches that may later be applied in less constrained disease settings, where specificity is more difficult to achieve.

### 4.6. Oncology: Specificity, Clinical Realism, and Multimodal Integration

Cancer has long been regarded as one of the most promising areas for breath diagnostics, yet it is also among the most difficult in which to establish specificity. The problem is not simply whether altered VOC patterns can be detected, but what those patterns actually reflect. Oxidative stress, inflammation, treatment effects, smoking exposure, and microbiome-related changes can all contribute to the breath profile of a patient being assessed for cancer. None of these processes is unique to malignancy, and all are common in the at-risk populations in which cancer is most likely to develop. For that reason, confounding does not arise as a separate problem after the fact. It is part of the clinical pathway itself. A given breath signal may therefore reflect tumor biology, the effects of therapy, or background comorbidity. Metabolic reprogramming is a plausible contributor to some signatures. Altered central carbon metabolism and mitochondrial function can shift ketone handling and redox balance, which in turn can influence acetone and carbonyl-related readouts. Changes in lipid turnover and in isoprenoid synthesis may also contribute to aldehyde profiles and isoprene dynamics. However, these pathway-level changes overlap with those of infection and chronic inflammatory disease, making specificity a design problem rather than an analytical one. This is why claims of disease specificity depend so heavily on careful comparator design and rigorous validation. The same concern has appeared repeatedly in the literature, as summarized in a meta-analysis of VOC-based breath tests, which identified ongoing methodological weaknesses, including variation in sampling methods and a lack of multicenter validation [79]. More recent reviews have described encouraging pooled performance but also point to substantial heterogeneity, the repeated observation of nonspecific VOCs, and the limited value of classical case–control designs in clinically realistic settings [80]. These studies suggest that the key issue in oncology is not whether a signal can be found, but whether that signal remains informative when tested against the differential diagnoses encountered in routine practice.

Markar et al. [81], Amal et al. [82], and Hong et al. [83] each moved oncology breath research away from simple case–control separation and toward more clearly defined diagnostic questions. Steenhuis et al. [84] applied the same line of thinking in colorectal cancer follow-up. In lung cancer, Steenhuis et al. [85] reviewed the electronic-nose literature and reported promising pooled sensitivity and specificity. Zhao et al. [86], in turn, examined malignant pleural mesothelioma against a comparator group of asbestos-exposed but asymptomatic individuals. Other studies have compared suspected lung cancer with COPD and related chronic inflammatory lung diseases [87,88,89]. These examples make the central point clear. The clinically relevant comparisons are not cancer versus health, but malignant versus benign nodules in smokers, cancer versus chronic inflammatory lung disease, and recurrence versus post-treatment inflammation. Each of these situations poses a different problem and therefore requires a different balance between sensitivity, specificity, and tolerance of false-positive results. Breath is also more likely to be accepted in oncology when it is used as part of a broader evidence framework rather than as a stand-alone signal. This is already reflected in work combining breath metabolites with imaging and routine clinical variables, where multi-omics fusion improved discrimination in the assessment of breast lesions [90]. Interest is also increasing in more complex AI models designed to learn hierarchical breath representations [91]. Their promise, however, should be interpreted cautiously. Their clinical value will depend on locked development, strict external holdout sets, and comparator groups that represent real diagnostic uncertainty. Without these safeguards, such models may reflect clinical site environmental features, sampling routines, or prevalence differences rather than disease biology. For that reason, the most credible translational route in oncology is likely to involve AI-supported data fusion, multicenter external validation, transparent subgroup reporting, and study designs anchored to meaningful clinical outcomes. In vitro headspace studies remain useful, but mainly as a source of mechanistic hypotheses. VOC mixtures released from tissue may separate under controlled conditions, yet this cannot replace in vivo validation in clinical populations [92]. More than any other application area, oncology demonstrates why breath analysis should be incorporated into a broader diagnostic framework rather than presented as a definite answer in isolation.

## 5. Assay Standardization, Metrology, and Model Robustness

For a breath-based test to be clinically useful, several conditions must be met from the outset. Sampling must be controlled, confounding factors must be measured explicitly, the analytical chain must be traceable, and the resulting models must remain reliable when used outside the study in which they were developed. The field is no longer limited by a lack of measurable chemistry. A number of platforms can now generate rich, time-resolved readouts and, under controlled conditions, distinguish between clinical groups. The more difficult question is whether the same result remains stable when the method is used across different patients, rooms, seasons, devices, and clinical workflows. That is why reproducibility has become central to translation. In other words, breath analysis is shaped less by analytical sensitivity alone than by factors that occur before and around the measurement, including pre-analytical handling, control of confounding factors, and the way the model is built and maintained. The attraction of breath analysis lies in how closely it reflects physiology and environment; however, this is also what makes it vulnerable. The measured signal depends on how the subject breathes, which fraction of the breath is sampled, the recent history of the inlet, and what the subject has inhaled or ingested shortly beforehand.

### 5.1. Standardized Sampling and Breath Fraction Control

Breath fraction is a key parameter and should not be treated as a minor technical point [13]. Dead-space air, end-tidal air, airway aerosol, and condensate microdroplets do not represent the same material, either chemically or biologically, and the end result changes accordingly. Even so, terms such as late expiratory, end-exhaled, and alveolar are still used inconsistently in the literature, which makes comparison between studies more difficult than it should be. For this reason, fraction control needs to be planned deliberately and described in enough detail for the protocol to be reproduced elsewhere. The sampling method should explain exactly how the patient breathes during collection, including whether the sample is taken during normal breathing or a deep breath, whether a breath hold is used, and whether exhalation is performed against resistance. It should also make clear how the relevant phase of exhalation is selected, whether by CO_2_ gating, flow or pressure triggers, or buffered end-tidal capture. The interface used for collection also matters. Mouthpieces, masks, and non-contact arrangements each distinctly affect dilution and contamination. Acceptance criteria are therefore essential. These usually include the quality of the CO_2_ plateau, checks for leaks, and a minimum exhaled volume. When these conditions are not met, the sample is better discarded than corrected afterward, since post hoc correction can shift the analysis away from biology and toward artifact. Breath sampling can remain easy for patients and practical in routine care, but it can only be standardized if the collection process is properly instrumented [27,28,29]. Non-contact and mask-based systems reduce the amount of coaching required, yet they are also more vulnerable to dilution and to interference from room air. Under those circumstances, paired room-air sampling and breath-cycle metadata become part of the specimen rather than optional background information [93]. The same applies when particle-associated biomarkers are being studied. In that setting, the patient’s breathing pattern and the sampling setup should both be recorded, because the number of particles in exhaled breath can be influenced by airway behavior and by how the sample is collected [24]. Put simply, if two centers cannot reproduce the same breathing pattern under the same instructions and acceptance criteria, they are not performing the same assay, even if they use the same detector and the same model.

### 5.2. Confounding Variables as Measured Assay Parameters

Several of the factors discussed in Section 2.4 are better regarded as part of the assay than as background noise to be filtered out. Ambient exposure, recent diet, oral chemistry, smoking, and time of day can all shape the measured breath profile [5,6,25,26]. For that reason, it is usually better to record these variables explicitly and account for them in the analysis than to deal with them only by excluding participants later. In practical terms, this starts with the sampling itself. Breathing mechanics should be documented, including flow, CO_2_ profile, confirmation of end-tidal sampling, and the quality of the breathing action during sampling. The surrounding environment also needs attention. Where possible, room air should be sampled alongside breath, and relevant details such as ventilation conditions or obvious exposure events should be noted. The same applies to the participant’s immediate physiological state. Food intake, smoking or vaping, medication timing, recent activity, stress, and pain may all influence the resulting profile and should be considered during both study design and interpretation. The choice of comparator group is part of the same problem. Control groups should reflect the clinical setting in which the test is meant to be used, rather than relying mainly on healthy volunteers. This becomes particularly evident in larger screening studies, where breath-based classification performs well only when differences in sampling conditions, participant background, and site-specific practice are built into the assay instead of being treated as an afterthought [36]. Confounding, then, is not just a statistical nuisance; it is part of the circumstances under which breath is produced, collected, and interpreted, and it needs to be measured on those terms.

### 5.3. Metrology, Biomarker Identification, and Quality Control

Analytical capability alone is not enough to establish a clinically useful breath test. A breath-based system also requires a measurement framework that defines what is being measured, how calibration is performed, how drift is monitored, and how results are kept comparable across instruments and over time [38,39,42]. In practice, three recurring metrology issues determine whether a reported breath signature can be relied upon beyond a single study. The first concerns reactive and surface-active VOCs. These compounds are particularly susceptible to distortion during sampling and analysis. Inlet materials, humidity, ion chemistry, and memory effects can all alter their apparent abundance and, in doing so, change the feature set presented to the model. This matters because aldehydes and sulfur-containing compounds are often linked to oxidative stress and inflammation, yet these same pathways are not specific to a single disease state, particularly in oncology. Studies that have improved inlet design and calibrated measurements under humid breath-like conditions have shown that quantitative performance can be strengthened, but they also make a broader point. Reactive VOCs should be treated as a metrology problem before they are treated as a modeling problem [37]. The second issue concerns untargeted breath signatures. These broad signal patterns are useful in discovery studies, but they are less reliable as clinical endpoints unless the key contributing features are stable, well-defined, and reproducible. Many untargeted studies report mass-to-charge features, putative formulas, or multivariate pattern vectors. This is acceptable at an early stage, when the aim is to identify candidate panels. It becomes less satisfactory when those same features are intended to support clinical decisions. At that stage, stable feature definitions are required, along with internal references where feasible and periodic orthogonal confirmation of features that are expected to carry diagnostic or monitoring value. The short-chain fatty acid work provides a useful example of this type of confirmation strategy [40]. The third issue is quality control. In breath diagnostics, quality control cannot be added at the end of the workflow. It has to be built into both the device and the analytical process from the outset. This includes blanks, ambient references, drift sentinels, and performance control mixtures, together with acceptance limits that can stop reporting when measurements fall outside the calibrated range. Without these safeguards, models may fail quietly, particularly in distributed settings where operators, environments, and routines differ from site to site. For this reason, the translational pathway is likely to remain tiered. Untargeted fingerprinting may continue to play an important role in discovery, but clinical implementation will depend on features that are calibrated, traceable, and interoperable whenever the underlying chemistry allows it.

### 5.4. Bioinformatics, Overfitting, and Model Governance

Breathomics is particularly prone to overfitting, meaning a model may appear accurate on the development dataset but perform poorly when tested in new patients or in different clinical settings. Breathomics datasets often contain many measured features, while the number of patients is relatively small. Because the signal also changes with time and context, this creates a high risk of unstable models and overfitting. Under these conditions, AI modeling does not necessarily define disease. It may instead end up defining where and how the samples were collected. Clinic-specific room air, inlet history, cartridge batches, operator technique, and small differences in breathing maneuvers can all become embedded in the data, especially when cases and controls are not collected under the same workflow or within the same period. The risk of data leakage is practical and immediate, because models may otherwise learn features of the collection setting rather than the underlying biology. Even among healthy participants, breath profiles can retain stable personal and contextual structure. In one SESI-HRMS dataset, 227 features were sufficient to identify 28 of 31 participants, while 37 features varied with time of day [26]. If a spectrum contains enough information to indicate who was sampled and when, it may also contain enough information to reveal where and how the sample was obtained, unless the study design actively prevents this. This is particularly important in oncology, where effect sizes may be modest and confounding is common. In that setting, controlling leakage should be treated as a design requirement no less important than calibration of the assay itself. Instrumental drift and batch effects add a further layer of difficulty. They can change apparent discrimination without reflecting any true biological difference. Reported AUROC values have been shown to vary by approximately 0.1–0.2, depending on the preprocessing strategy and the choice of drift or batch correction applied to the same dataset [94]. In breathomics, the meaning of batch extends beyond a single analytical run. It may also reflect room-air composition, seasonal exposure patterns, inlet conditioning, maintenance schedules, staffing changes, or shifts in protocol. If these boundaries are ignored during validation, a model may separate the collection context rather than the physiology. For this reason, clinical breath bioinformatics should rely less on random data splits and more on validation strategies that test generalizability directly. Cross-site and cross-period holdouts should become routine. Data splits should respect batch structure, for example, through site-level or time-blocked evaluation. Feature selection and analysis plans should be defined in advance whenever possible, and models should be locked before prospective testing in cohorts that reflect the intended workflow. Drift and batch surveillance should also be built into the analytical pipeline itself, with quality control rules and acceptance thresholds that indicate when an instrument or a deployment has moved outside its calibrated operating range.

The use of AI is becoming increasingly important in real-time breath diagnostics. Most breath-analysis platforms generate multivariate spectra, and these are influenced not only by disease-related chemistry but also by humidity, breath fraction, flow, and background room air, all factors that should be measured and controlled. In PTR-ToF-MS and related workflows, machine-learning methods have already been used to convert large ion-feature datasets into rapid screening outputs in clinical settings [8,36]. This shows that algorithmic analysis can operate on timescales relevant to triage, provided that sampling and labeling are carefully controlled. Breath, however, is not a static specimen. It is a time-structured signal, meaning that kinetic information can improve interpretation by helping distinguish endogenous production from inhaled contaminants and by revealing responses to intervention that may be obscured when spectra are averaged. In practice, model performance is often shaped before classification is even attempted. Quality control and preprocessing steps are therefore critical. These include selecting the appropriate breath phase, confirming end-tidal sampling using CO_2_ structure, and parallel sampling of ambient air. Such steps reduce the risk that a classifier will rely on dead-space composition or exposure carryover rather than on physiological information. Questions of governance and privacy are also directly relevant. Multicenter learning will be necessary if breath models are to generalize across settings, but breath profiles may still be indirectly identifiable through stable personal patterns or unusual exposures. Federated learning and privacy-first analytical approaches offer one way to build and test models across sites without pooling raw patient-level data [95,96]. Their value, however, depends on transparent reporting of performance across sites, demographic groups, comorbidities, and collection environments. For this reason, breath models should not be viewed as fixed outputs of a single study. They should be treated as clinical assets that require ongoing maintenance. Seasonal exposure drift, workflow changes, and instrument aging are all expected. If updates are made informally or not documented at all, performance loss is likely to be gradual, difficult to detect, and clinically important.

### 5.5. Regulatory Pathways for Device–Algorithm Systems

**Figure 4:**
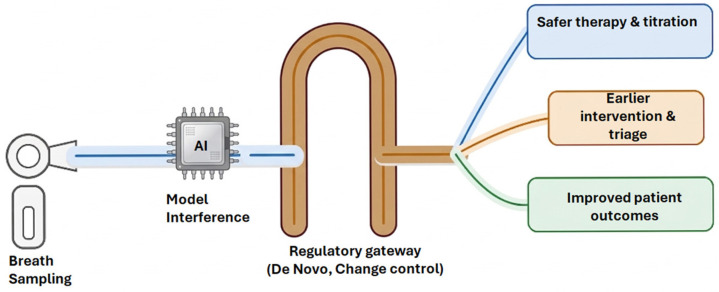
Regulatory pathway for breath diagnostic device-and-algorithm systems. This process encompasses intended use, risk classification, evidentiary pathway, algorithm change control, and validation linkage across the product lifecycle.

Many clinically useful breath-based systems will combine a physical device with software that can be updated over time. This creates a regulatory challenge that extends beyond analytical performance alone. There is already precedent in breath diagnostics from FeNO systems, where exhalation control, operating conditions, and quality control are built into the device itself. For newer breathomics platforms, particularly those without a clear predicate device, the regulatory route is more likely to follow a risk-based framework [97]. The issue becomes more complicated when the product includes an algorithm that contributes directly to the result, because in that setting the software is not simply a reporting tool but part of the diagnostic function. Two regulatory points follow from this. First, the intended use of the system determines the level of evidence required. A general wellness claim falls within a lower-risk, non-diagnostic category, whereas a diagnostic or therapy-guiding claim places the system within medical device regulation and requires evidence that matches that purpose [98]. Second, any future model updates need to be anticipated and managed in advance. Predetermined change-control plans are intended to make such updates part of a formal regulatory process rather than an informal technical adjustment [99]. This is particularly relevant in breath diagnostics, where dataset shift is likely to occur as devices age, workflows change, and exposure patterns vary over time. For that reason, regulatory planning needs to begin early and develop in parallel with the measurement strategy and inference pipeline. Quality systems, drift control, auditability, and change management are not secondary administrative matters, but part of the product itself. Figure 4 outlines this device-and-algorithm pathway and shows how it links to validation.

### 5.6. Outcome-Based Validation and Clinical Implementation

Accuracy, sensitivity, and AUC describe how well a system separates groups, but by themselves, they do not show that the result is clinically useful. For breath testing to have value in practice, it must improve clinical management. This may involve shortening the time to isolation decisions in syndromic respiratory illness, prompting earlier escalation when deterioration is genuine, supporting de-escalation when confirmatory testing is negative, reducing exacerbations through earlier treatment, or improving stewardship by helping distinguish inflammatory relapse from noninflammatory symptoms. Validation should therefore extend beyond diagnostic performance and examine what happens downstream when the test is used under routine clinical conditions. Relevant outcomes include decreased time to decision, days of isolation avoided, antibiotic exposure, admissions, ICU transfers, exacerbation frequency, length of stay, and the consequences of false-positive and false-negative results. Wherever possible, such studies should be prospective, embedded in the clinical pathway in which the test is intended to operate, and follow prespecified analysis plans. Claims of implementation also require evidence that the system remains reliable outside a single center or a tightly controlled study. Breath-based platforms need to show that measurements are reproducible across sites, seasons, operators, and devices, with clear reporting of sampling adherence, ambient conditions, and quality control failures. This is particularly important because the clinical value of breath testing may be greatest in settings where conventional laboratory turnaround is difficult to achieve. In this respect, breath diagnostics should be viewed not only as an analytical task, but also as an implementation task. Sampling, metrology, inference, governance, and clinical treatment all need to be developed together and evaluated as parts of the same assay. The validation pathway for breath biomarkers also differs from that of more established diagnostic modalities. Imaging biomarkers are usually linked to lesion localization, standardized acquisition methods, and familiar interpretive frameworks. Liquid-biopsy markers, by contrast, often rely on defined molecular targets that can be compared directly with tissue pathology or genotyping. Breath biomarkers rarely have either of these advantages but are more susceptible to pre-analytical variability, environmental carryover, and dependence on multivariate patterns. Their strength lies in frequent, non-invasive sampling, which enables the tracking of physiological changes over short timescales. For that reason, validation in this field should draw on the discipline used for established biomarker pathways, while giving particular attention to issues that are specific to breath, such as breath fraction control, measurement of confounders, robustness in longitudinal use, and clinical utility defined in relation to the intended use.

## 6. Future Directions: Integration into Clinical Workflows

**Figure 5:**
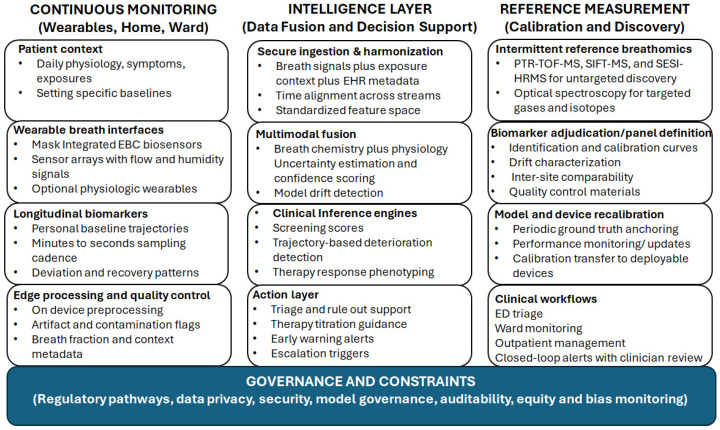
An integrated future architecture for breath-based diagnostics and monitoring. This conceptual systems diagram links wearable longitudinal monitoring, intermittent reference measurement, multimodal inference, and clinical decision outputs within regulatory and privacy constraints.

Once validation has been aligned with intended use, the next challenge is implementation in routine care. Future progress will depend not only on the ability to acquire repeated breath measurements, but also on whether those measurements can be translated into decisions reliable enough to guide effective clinical action. Figure 5 summarizes this logic. As discussed by Rattray et al. [100], the value of repeated sampling lies in its ability to follow temporal change rather than depend on isolated measurements. For this to be useful in practice, breath systems will need individualized baselines, structured incorporation of breathing patterns and exposure metadata, and clear rules for handling measurements obtained under unstable or poorly calibrated conditions. The resulting outputs will also need to be presented in a form that clinicians can act upon, for example, as a probability of deterioration or recovery linked to recommendations for repeat sampling, confirmatory testing, escalation, or continued monitoring. When uncertainty is high, this should be stated explicitly rather than masked by an apparently confident label. A further issue is dataset shift. In breath diagnostics, seasonal exposures, device aging, inlet history, and differences in workflow can all alter the measured feature space even when the underlying biology has not changed. This means that quality control, calibration transfer, and drift surveillance will need to be incorporated into routine lifecycle management rather than treated as occasional technical checks. This heightens the need for more advanced and complete electronic health records, and for artificial intelligence electronic health systems capable of highlighting and integrating disparate data sources over time to support clinical action, rather than simply applying machine learning to datasets in isolation. Finally, multicenter learning will be necessary if breath systems are to generalize across settings, but this also raises privacy concerns, particularly for wearable platforms that generate dense longitudinal profiles.

Alongside efforts to stabilize endogenous breath signatures, induced volatolomics is emerging as a route to pathway-specific readouts. In this approach, a defined exogenous substrate is administered, and the appearance or clearance of a volatile reporter in breath is measured. The exogenous volatile organic compound concept frames these probes as functional assays of selected metabolic routes, shifting interpretation from associative fingerprints to enzyme-linked chemistry [101]. In cancer models, substrate design has focused on tumor-associated enzymes, such as extracellular beta-glucuronidase, which cleave a glucuronide precursor to release a labeled volatile, including deuterated ethanol, that can be separated from background breath and tracked in real-time [102]. Related strategies based on volatile-releasing nanosensors extend induced readouts to protease activity in the lung, enabling breath-based monitoring of airway inflammation and response dynamics [103]. Translation will require the same sampling and metrology discipline used for endogenous breathomics, together with pharmacology-specific constraints such as dose selection, kinetic characterization, and careful bounding of off-target metabolism and microbiome contributions. Regulatory evaluation is also likely to resemble a drug-device combination, in which the probe, sampling interface, and algorithm are assessed as a single assay.

Several recent reviews have cataloged candidate breath biomarkers across broad disease areas and in oncology-focused settings [104,105,106]. The present review is intended to complement these syntheses by concentrating on real-time measurement and on the molecular logic that makes breath actionable. We emphasize how pathway-level processes map to specific analytes, how kinetic structure and fraction control stabilize interpretation, and how clinical deployment depends on metrology, model governance, and decision-anchored validation rather than on classification alone.

Recent work also shows that induced volatolomics can be paired with portable sensing. Phenyl-β-D-glucuronide has been used as an administered substrate that is hydrolyzed by tumor-associated β-glucuronidase to release phenol, which can then be measured in exhaled breath using a handheld analyzer. This type of activatable chemistry illustrates how pathway-anchored breath testing may reduce the specificity burden of endogenous profiles, but it will still require dose and kinetic characterization, as well as clinically faithful comparator sets [107]. In parallel, AI-powered nanosensor platforms are being developed to translate multiplexed breath signatures into point-of-care decisions, yet their clinical value will depend on calibration traceability, drift surveillance, and transparent model governance [108].

## 7. Conclusions

Breath diagnostics are most likely to enter clinical practice first in settings where sampling can be standardized, comparator groups reflect real clinical differentials, and the resulting readout can be linked to a clear clinical response. At present, these conditions are best met in respiratory infection triage, peri-procedural monitoring, dialysis-linked metabolic assessment, and the longitudinal management of inflammatory airway disease, where timing matters and sampling interfaces are relatively stable. The main barrier to wider clinical use is no longer the ability to detect breath signals, but the ability to obtain measurements that remain reproducible across different sites and workflows. From a molecular perspective, the strongest breath claims will be those that link signals to defensible biochemical provenance and transport. Examples include acetone as a readout of ketone handling, isoprene as a physiology-sensitive product of isoprenoid metabolism, ammonia as a proxy for urea and nitrogen handling in renal impairment, nitric oxide as an airway signal tied to inducible nitric oxide synthase activity in type 2 inflammation, and reactive carbonyls as downstream products of lipid peroxidation during oxidative stress. Because many of these pathways converge across diseases, breath signatures should be framed as biochemical states rather than as single-molecule disease labels, and key discriminatory features should be supported by traceable metrology, appropriate calibration in humid matrices, and orthogonal confirmation when molecular identity is central to interpretation. Progress will therefore depend on standardized breath fraction control, clinically realistic study design, pathway-anchored feature definitions, and appropriately governed devices and algorithm systems that support better decision-making rather than simply separate groups. Under these conditions, breath analysis may shift from a promising research tool to a reliable clinical assay that reports molecular physiology at the time scale of care delivery.

## Figures and Tables

**Table 1:** Representative breath analytes and analyte classes detectable in real-time platforms: chemical class, dominant origin, clinical contexts, platforms, and key translational considerations.

Analyte or Analyte Class	Chemical Class	Dominant Origin or Pathway	Most Credible Clinical Contexts	Best-Fit Real-Time Platform(s)	Key Translational Caution or Interpretation
Acetone	Ketone	Systemic fatty-acid oxidation and ketone handling	Metabolic monitoring, exercise physiology, diabetes, ketoacidosis	PTR-ToF-MS, SIFT-MS, chemiresistive sensor arrays	Strong real-time signal and deployable-sensor relevance, but strongly influenced by fasting status, diet, and exertion
Isoprene	Hydrocarbon terpene	Systemic isoprenoid metabolism with strong perfusion and ventilation dependence	Exercise physiology, perfusion-linked monitoring, peri-procedural dynamics	PTR-ToF-MS, SIFT-MS	Best interpreted as a dynamic physiology signal rather than a disease-specific biomarker
Ammonia	Inorganic gas	Systemic urea and nitrogen handling, with oral contribution	Renal failure, dialysis adequacy, uremia, serial metabolic monitoring	IMS or GC-IMS, SIFT-MS, CRDS, targeted sensors	Among the most physiologically interpretable breath markers, but oral chemistry, humidity, and ambient contamination must be controlled
Nitric oxide (FeNO)	Inorganic gas	Airway epithelial iNOS and type 2 inflammatory signaling	Asthma phenotyping, steroid response monitoring, selected chronic airway disease	Targeted FeNO analyzers, chemiluminescence, electrochemical or optical platforms	Already clinically standardized, but reflects inflammatory endotype more than disease label
Aldehydes and related carbonyls (e.g., hexanal, nonanal)	Aldehydes and carbonyls	Airway and systemic lipid peroxidation and oxidative stress	Oxidative injury, inflammatory phenotyping, adjunct infection or oncology panels	PTR-ToF-MS with careful inlet control, SESI-HRMS, orthogonal GC-MS confirmation	High information content, but reactive species are vulnerable to inlet memory, humidity effects, and surface losses
Short-chain fatty acids (acetate, propionate, butyrate)	Carboxylic acids	Gut microbial fermentation with systemic appearance in breath	Gut–lung axis studies, diet–microbiome response, metabolic inflammation	SRI-ToF-MS or PTR-based MS with TD-GC-MS confirmation	Mechanistically attractive, but strongly diet-responsive and usually requires structural confirmation
Nitrite and hydrogen peroxide in EBC	Nitrosative and oxidative airway-surface markers	Airway lining fluid and condensate-phase inflammatory chemistry	Longitudinal airway inflammation and oxidative-stress monitoring, wearable EBC workflows	EBC microfluidic and electrochemical sensing platforms	Useful for repeated airway monitoring, but dilution normalization, salivary contamination, and collection consistency are critical

**Table 2:** Comparative analysis of real-time breath analytical platforms: performance metrics and clinical fit.

Platform Family	Primary Targets	Typical Time Resolution	Deployment Setting	Main Translational Limitation	Best Fit Clinical Use	Key Examples
PTR-MS and ToF variants (PTR-MS, PTR-ToF-MS, CI-ToF-MS)	Broad VOC patterns; oxygenated and some reactive VOCs with inlet control	0.1 to 1 s	Reference grade instruments in clinical studies	Cost and complexity; structural assignment often needs confirmation; humidity and inlet effects can bias reactive VOCs	Discovery and calibration tier; high throughput screening where kinetics add value	[8,12,32,36]
Sampling and breath fraction control interfaces (BET, CO_2_ gating, noncontact, mask inlets)	Breath fraction definition (end tidal vs. mixed); maneuver quality metrics	Breath by breath	Cross-cutting pre-analytical layer	Adds hardware and protocol discipline; noncontact designs increase dilution and ambient sensitivity	Essential enabler for cross-site comparability and model robustness	[12,61]
SIFT-MS	Targeted VOCs and small gases with direct quantification	Seconds	Bedside capable in structured environments	Limited chemical breadth; depends on predefined analytes and sampling line handling	Operating room and ICU monitoring; targeted panels requiring absolute values	[43]
SESI-HRMS and related ambient ionization HRMS	Untargeted polar and semi-volatile metabolites; high-dimensional fingerprints	Seconds	Reference grade discovery	Context sensitivity and identification burden; strong leakage risk without rigorous design	Mechanistic discovery; response phenotyping; individualized baseline studies	[24,25,26,45]
IMS and GC-IMS	Constrained VOC panels; small polar gases such as ammonia	Tens of seconds to minutes	Point-of-care oriented	Humidity and matrix sensitivity; drift and library matching constraints	Targeted screening and monitoring in structured workflows	[46]
Optical spectroscopy (frequency comb, CRDS, photoacoustic, mid IR)	Selected small gases and isotopologues, especially CO_2_; tracer-based physiology	Seconds to minutes	Specialized reference layer	Limited analyte set tied to absorption lines; photonic complexity	Reference quantification; isotope physiology; calibration anchor for hybrid systems	[7,46,51]
Chemiresistive sensor arrays and electronic noses (MOX, graphene MOX, doped oxides)	Priority gases and pattern signatures (acetone, ammonia, mixed VOC surrogates)	Seconds	Wearable or low-cost edge	Drift, humidity interference, and cross-sensitivity; interpretability limitations	High frequency monitoring and screening adjuncts where usability dominates	[53,54,55,57]
Resonant and breath pattern transducers (QCM, magnetoelastic, fiber, and textile sensors)	Humidity and breathing pattern; adjunct gating and adherence	Sub-second to seconds	Wearables	Limited chemical specificity without added functional layers	Breath maneuver QC, adherence and physiologic context for chemical sensing	[56,58,59,60]
EBC microfluidics with electrochemical biosensing (mask platforms)	Nonvolatile ions and selected inflammatory or redox markers	Minutes	Wearable monitoring	Dilution normalization; oral contamination control; fouling and carryover	Longitudinal airway inflammation and oxidative stress monitoring	[61,62,63]
Breath aerosol and particle-phase analysis (online)	Particle-borne and microdroplet-associated analytes, including nonvolatile species	Seconds	Research and mechanistic studies	Strong dependence on particle generation and sampling geometry	Mechanistic transport studies and pharmacokinetic applications	[24]

**Table 3:** Key validation and translational studies across disease areas and platform types.

Disease Area/Use-Case	Platform and Modality	Cohort and Setting	Key Performance/Findings	Translational Note	Study
ACUTE INFECTIOUS DISEASE TRIAGE					
COVID-19 screening	PTR-TOF-MS breathomics + machine learning + metadata	173 participants (67 COVID-19 positive)	Sensitivity 98%, specificity 74%, PPV 72% NPV 98% AUC 0.961	Supports rapid front-end screening in prospective hospital triage; metadata integration improved performance	[8]
COVID-19 biomarker identification and screening	PTR-TOF-MS + machine learning framework	1137 participants; independent test cohort n = 340	Accuracy 81.2% overall; 97.3% in participants > 55 years	Overlap with influenza-like breathprints + ion-level limitations highlight need for realistic differentials	[36]
Ventilator-associated lower respiratory tract infection	Untargeted GC-MS breath analysis	108 invasively ventilated ICU patients with suspected VA-LRTI	Sensitivity 98% at specificity 49%; NPV 96%	Strong rule-out potential in ventilated ICU workflows; supports breath as an adjunct to antimicrobial decision-making in suspected VA-LRTI	[64]
RESPIRATORY DISEASE AND AIRWAY BIOCHEMISTRY					
Continuous airway and metabolic monitoring	Mask-based EBC harvesting + electrochemical biosensors	Controls + COPD/asthma/post-COVID groups	EBC nitrite vs. FeNO r = 0.795; EBC ammonium vs. serum urea r = 0.846	Minute-scale workflow; validated under real-world activity	[61]
Pharmacometabolomics; bronchodilator responsiveness	SESI-HRMS breath analysis	34 pediatric asthma patients; 38 visits	333 features altered post-salbutamol; metabotypes linked to poor responsiveness	Demonstrates response phenotyping embedded in outpatient workflow	[45]
METABOLIC PHYSIOLOGY AND GUT-LINKED METABOLITES					
Exercise stress testing (CPET)	SESI-HRMS	13 healthy adults (7 female)	33 metabolites change across intensity: pathway-level shifts (e.g., TCA/tryptophan)	Supports dynamic cardiometabolic phenotyping and rehab monitoring	[65]
SCFA monitoring; gut-linked volatiles	SRI-ToF-MS + TD-GC-MS confirmation	Method characterization + breath demonstration (humid/dry)	SCFA repeatability < 15% (acetic/propionic/butyric); structural confirmation by TD-GC-MS	Establishes analytical credibility; underscores orthogonal confirmation + standardized sampling	[40]
PERIPROCEDURAL AND CRITICAL CARE MONITORING					
Perioperative monitoring (anesthesia/ventilation)	Online SIFT-MS integrated into the ventilation circuit	5 anesthetized patients	Propofol + acetone/isoprene/water vapor; rapid responses to operative events	Demonstrates feasibility integrated into existing clinical infrastructure	[43]
RENAL AND UREMIC METABOLISM MONITORING					
Dialysis adequacy; uremic metabolism	IMS + cavity ring-down spectroscopy	20 hemodialysis + 20 controls; serial sampling	Breath ammonia decreases during dialysis; correlates with BUN and Kt/V	Clear monitoring use case with interpretable physiology and actionability	[46]
ONCOLOGY (EARLY-STAGE EXEMPLAR)					
In vivo validation in thoracic oncology outpatients	Cloud-connected eNose analysis of exhaled breath	364 adults with clinical and/or radiological suspicion of lung cancer from 2 thoracic oncology outpatient clinics; external validation cohort n = 121	Validation ROC-AUC 0.83; sensitivity 94%; specificity 63%; PPV 79%; NPV 89%; performance consistent across tumor characteristics, disease stage, centers, and clinical characteristics	Multicenter prospective external validation in a clinically realistic referral population, aligned with intended-use deployment	[66]
CROSS-CUTTING ENABLERS					
Healthy baseline variability mapping	SESI-HRMS + bioinformatics curation	31 participants	227 features uniquely identify 28/31; 37 signals associated with time of day	Justifies individualized baselines + diurnal control in models	[26]

## Data Availability

No new data were created or analyzed in this study. Data sharing is not applicable to this article.

## Abbreviations

AI	artificial intelligence
AUC	area under the curve
AUROC	area under the receiver operating characteristic curve
BET	buffered end-tidal
BUN	blood urea nitrogen
CI-ToF-MS	chemical ionization time-of-flight mass spectrometry
CMOS	complementary metal-oxide-semiconductor
CO	carbon monoxide
CO_2_	carbon dioxide
COPD	chronic obstructive pulmonary disease
COVID-19	coronavirus disease 2019
CPET	cardiopulmonary exercise testing
CRDS	cavity ring-down spectroscopy
EBC	exhaled breath condensate
FeNO	fractional exhaled nitric oxide
FDA	U.S. Food and Drug Administration
GC	gas chromatography
GC-IMS	gas chromatography–ion mobility spectrometry
HRMS	high-resolution mass spectrometry
ICU	intensive care unit
IMS	ion mobility spectrometry
Kt/V	dialysis adequacy index (dialyzer clearance × dialysis time/volume of distribution of urea)
MOX	metal oxide
MS	mass spectrometry
NO	nitric oxide
NPV	negative predictive value
PCB	printed circuit board
PMA	premarket approval
PPV	positive predictive value
PTR	proton transfer reaction
PTR-MS	proton transfer reaction mass spectrometry
PTR-ToF-MS	proton transfer reaction time-of-flight mass spectrometry
QC	quality control
QCM	quartz crystal microbalance
SCFA	short-chain fatty acid(s)
SESI	secondary electrospray ionization
SESI-HRMS	secondary electrospray ionization high-resolution mass spectrometry
SIFT	selected ion flow tube
SIFT-MS	selected ion flow tube mass spectrometry
SRI-ToF-MS	selective reagent ionization time-of-flight mass spectrometry
TCA	tricarboxylic acid
TD-GC-MS	thermal desorption gas chromatography–mass spectrometry
ToF	time-of-flight
VOC	volatile organic compound
VOCs	volatile organic compounds
